# Construction of a high-density, high-resolution genetic map and its integration with BAC-based physical map in channel catfish

**DOI:** 10.1093/dnares/dsu038

**Published:** 2014-11-26

**Authors:** Yun Li, Shikai Liu, Zhenkui Qin, Geoff Waldbieser, Ruijia Wang, Luyang Sun, Lisui Bao, Roy G. Danzmann, Rex Dunham, Zhanjiang Liu

**Affiliations:** 1 The Fish Molecular Genetics and Biotechnology Laboratory, School of Fisheries, Aquaculture, and Aquatic Sciences and Program of Cell and Molecular Biosciences, Aquatic Genomics Unit, Auburn University, Auburn, AL 36849, USA; 2 USDA-ARS Warmwater Aquaculture Research Unit, Stoneville, MS 38776, USA; 3 Department of Integrative Biology, University of Guelph, Guelph, Ontario N1G 2W1, Canada

**Keywords:** SNP, catfish, linkage map, physical map, genome

## Abstract

Construction of genetic linkage map is essential for genetic and genomic studies. Recent advances in sequencing and genotyping technologies made it possible to generate high-density and high-resolution genetic linkage maps, especially for the organisms lacking extensive genomic resources. In the present work, we constructed a high-density and high-resolution genetic map for channel catfish with three large resource families genotyped using the catfish 250K single-nucleotide polymorphism (SNP) array. A total of 54,342 SNPs were placed on the linkage map, which to our knowledge had the highest marker density among aquaculture species. The estimated genetic size was 3,505.4 cM with a resolution of 0.22 cM for sex-averaged genetic map. The sex-specific linkage maps spanned a total of 4,495.1 cM in females and 2,593.7 cM in males, presenting a ratio of 1.7 : 1 between female and male in recombination fraction. After integration with the previously established physical map, over 87% of physical map contigs were anchored to the linkage groups that covered a physical length of 867 Mb, accounting for ∼90% of the catfish genome. The integrated map provides a valuable tool for validating and improving the catfish whole-genome assembly and facilitates fine-scale QTL mapping and positional cloning of genes responsible for economically important traits.

## 1. Introduction

Genetic linkage maps are essential for the understanding of genomic levels of organization of inheritance of traits.^1^ Construction of high-density and high-resolution genetic maps is a key step for fine mapping of quantitative trait loci (QTL) and marker-assisted selection. In addition, genetic linkage maps are valuable resources for the generation of chromosome-level assembly of whole-genome sequences and for comparative genome analysis.^2^ In most of the recent whole-genome sequencing cases, whole-genome sequences are generated using next-generation sequencing (NGS). Short sequence reads are assembled into contigs. Such contigs are generally still relatively short although they vary in sizes from several kilobases to tens of kilobases. Increases in genome sequencing coverage and sequencing libraries can increase the quality of the assembly, allowing the sizes of contigs to be increased. However, NGS methods alone cannot provide the resources to assemble complex genomes at the chromosomal level. Genome sequence assemblies at this level require the assembly of tens of thousands to hundreds of thousands of contigs.

Highly segmented genome assemblies prohibit efficient genome analysis. Therefore, various genome resources have been created to reduce the segmentation of the genome assemblies. One of these resources is the large-insert-based physical maps. Historically, several types of large insert libraries have been used. These include yeast artificial chromosomes (YACs),^3^ bacterial artificial chromosomes (BACs),^4^ and cosmid-based libraries.^5^ YACs have the largest capacity for cloning the large inserts, but they are relatively unstable; therefore, their use in genome studies has been limited. Cosmid libraries have the smallest capacity for cloning the large inserts; therefore, their use in large insert libraries has also been limited. BACs are the most popular large insert libraries as they are stable and can hold inserts of up to 200 kb. BAC-based physical maps can organize the entire genome into restriction fingerprint-based contigs. Such contigs are similar to the whole-genome sequencing contigs, but they are constructed using overlapping restriction enzyme fingerprints rather than overlapping sequences themselves for the whole-genome sequence assemblies. By analysis of restriction fingerprints of overlapping genomic clones of BAC inserts, the whole genome can be organized into a limited number of contigs, most often in thousands. For instance, the catfish physical maps had 3,307 contigs and 1,891 contigs.^6,7^ The integrated catfish physical map included over 2,500 contigs (unpublished).

Integration of physical maps and whole-genome sequence contigs allows the relationship to be established between the sequence-based contigs and the restriction fingerprint-based contigs, thereby reducing the levels of segmentation of the genome. One of the major applications of genetic linkage map is to integrate physical maps and whole-genome assemblies. Integration of genetic map with physical map is useful for understanding genomes from different dimensions and is essential for comparative genome analysis,^8^ fine-scale QTL mapping and positional cloning of genes responsible for performance and production traits.^9,10^ In aquaculture fish species, genetic maps have been constructed in a few species, such as Asian seabass,^11^ Atlantic salmon,^12^ half-smooth tongue sole,^13^ rainbow trout,^14^ common carp,^15^ and catfish.^2,9,16,17^ These maps harbour several hundred to a couple of thousands of markers, with which QTL for agriculturally important performance and production traits can only be mapped in large genomic regions.

Integrated maps have been developed in several aquaculture fish species using low-density genetic maps. In Atlantic salmon, in addition to 579 BAC contigs that were integrated into the linkage map using microsatellite markers, identification and mapping of new BAC-anchored single-nucleotide polymorphism (SNP) markers from BAC-end sequences placed 73 additional BAC contigs to Atlantic salmon linkage groups.^18^ The second generation of rainbow trout integrated map anchored up to 265 contigs to the genetic map, covering ∼11% of the genome.^10^ In common carp, a total of 463 physical map contigs and 88 single BACs were integrated into the genetic linkage map, which covered 30% of the common carp genome.^15^ In catfish, 2,030 BAC-end sequence (BES)-derived microsatellites from 1,481 physical map contigs were used for linkage map construction, which anchored 44.8% of the catfish BAC physical map contigs, covering ∼52.8% of the genome.^9^ Apparently, the level of integration is dependent on the density and resolution of the genetic maps.

One objective of this study was to construct a high-density and high-resolution genetic linkage map by using a large number of molecular markers, covering the entire genome and a large resource panel for linkage mapping analysis. In addition to this primary goal, a secondary goal was to increase integration of the linkage and physical maps, using markers derived from BECs for genetic linkage analysis. Our previous genetic linkage analysis used mostly microsatellite markers. In spite of their high polymorphism, and low cost of single marker genotyping, an analysis of tens of thousands of microsatellites with a large number of mapping fish is extremely labourious and cost-prohibitive. SNPs overcome these difficulties by providing high efficiency and low-cost, large-scale genotyping. SNPs have become markers of choice because of their abundance, even genomic distribution, and easy adaptation to automation.^19^ Recent advances in NGS have allowed rapid discovery of genome-wide SNPs in any organism in a cost-effective manner.^20^ With the availability of large numbers of SNPs, high-density SNP array platform can be developed for high-throughput and efficient genotyping. Alternatively, an NGS-based genotyping-by-sequencing (GBS)^19–21^ is also highly efficient. SNP arrays and GBS have both been used to genotype large-scale SNPs for genetic mapping and association analyses.^12,22–28^ Compared with GBS, SNP arrays are more cost-effective. In addition, they provide a greater level of genome coverage. For instance, in most cases, the total number of commonly analysed SNPs is limited to several thousands while millions of SNPs can be analysed by using very high-density SNP arrays. In addition, GBS of large resource panels for high-resolution maps is cost-prohibitive.

Channel catfish, *Ictalurus punctatus,* is the primary aquaculture species in the United States. Since the initiation of genome research over two decades ago, several genetic maps have been constructed with different types of molecular markers and various resource families.^2,9,16,17^ Up to date, the highest density map was developed to contain 2,030 microsatellites and 100 SNPs.^9^ Although this genetic map has been very useful for genetic and genomic analysis,^29,30^ marker density in this map was still fairly low and only facilitated integration to the physical map for ∼52% of catfish genome. Recently, following efforts to expand catfish genomic resources, we have identified millions of SNPs in channel catfish^31,32^ and developed high-density SNP arrays,^33^ which provided the opportunity to develop a high-density and high-resolution SNP-based genetic map. Here we report the construction of genetic linkage map with over 50,000 SNPs in channel catfish, which is, to the best of our knowledge, the highest density genetic map for any aquaculture species. Along with the high density of markers, the utilization of BAC-associated SNPs also allowed significant increase of the integration of the genetic linkage map with the BAC-based physical map in catfish, allowing over 90% of the catfish genome physical map contigs to be mapped to linkage groups. This genetic map should serve as a valuable framework for validating the reference whole-genome sequences, extensive comparative and functional genomic studies, and fine-scale QTL mapping and association studies in catfish.

## 2. Materials and methods

### 2.1. Resource families and DNA preparation

A total of 576 fish, with 192 full-sibling individuals from each of three full-sibling channel catfish families, were used for linkage mapping. The parent and grandparent samples were also obtained. All DNAs for these samples used in this study were provided by USDA-ARS Warmwater Aquaculture Research Unit, which were prepared following the procedures as previously described.^17^

### 2.2. SNP genotyping

DNA samples were arranged in 96-well microtitre plates and diluted to a final concentration of 50 ng/µl with the final volume of 10 µl. Genotyping was conducted by GeneSeek (Lincoln, NE, USA) using the catfish 250K SNP array.^33^ Affymetrix CEL files were analysed using Affymetrix Genotyping Console software (version 4.0) for quality control analysis and SNP genotype calling using the Affymetrix AxiomGT1 algorithm. Samples passing the quality control (Dish value > 0.85) and SNP call rates threshold (>95%) were retained for analysis. Following genotyping, an R package, SNPolisher, was used to generate the genotyping outputs. The package produced SNP quality control metrics and divided SNPs into several classes based on the quality of genotype calls.^33^ The polymorphic markers with high resolution of cluster separation (high genotyping quality) were remained for further analysis. The CHP files generated from the Affymetrix Genotyping Console were imported into SNP & Variation Suite (SVS version 7, Golden Helix, Inc.) for further filtering to remove SNPs with missing genotypes >10% and minor allele frequency <5%.

### 2.3. Linkage map construction

Only SNPs that had high quality of genotype calls and were heterozygous in at least one parent were retained for linkage analysis. Based on segregation patterns, SNPs were categorized into three types: 1 : 2 : 1 type (AB × AB, segregating in both parents), 1 : 1 type (AB × AA or AB × BB, segregating only in female), and 1 : 1 type (AA × AB or BB × AB, segregating only in male). The *χ*^2^ goodness-of-fit tests were performed to examine the segregation distortion. Markers with significant segregation distortion were excluded (*P* < 0.001).

Linkage maps were initially developed independently for the three mapping families. Sex-specific maps were constructed for each parent using 1 : 1 segregation-type markers only (AB × AA or AB × BB for female map and AA × AB or BB × AB for male map). The markers of segregation type 1 : 2 : 1 (AB × AB) were used to build the sex-averaged map. The LINKMFEX software (version 2.4, http://www.uoguelph.ca/~rdanzman/software.htm) was used to perform linkage analysis for sex-specific markers, and OneMap (R package, version 2.0) was used for linkage analysis of sex-averaged markers. Linkage between markers was examined by estimating logarithm of the odds (LOD) scores for recombination fraction (*θ*). A LOD threshold of 8.0 was used, and a maximum *θ* of 0.35 was set to assign markers into linkage groups.

For markers falling into ‘zero recombination clusters', only one marker was picked that had the most informative meiosis as the representative marker of each cluster for linkage map construction to reduce the power required for computation of linkage. Genetic maps were constructed using JoinMap software version 4.0 with the regression mapping algorithm.^34^ The Kosambi mapping function was used to convert the recombination frequencies into map distances (centiMorgans). The positions of markers were determined according to the sequential build-up of the map.^35^ Briefly, a pair of markers was firstly selected, followed by sequential addition of other markers. The ‘ripple’ was performed each time after adding one marker. The best fitting position of the added markers was searched based on the goodness-of-fit test for the resulting map. When a marker generated a negative map distance or a large ‘jump’ value in goodness-of-fit test, the marker was removed, and map calculation was continued to construct a first-round map. Thereafter, the removed markers were added to the first-round map and again subjected to the goodness-of-fit test to generate an optimum order of markers. For the sex-specific markers, the linkage phases were inferred automatically, while for the markers with 1 : 2 : 1 segregation type (AB × AB), the linkage phase was deduced based on the genotypes of grandparents to assist the map construction.

The consensus maps were then established using the MergeMap^36^ by integrating individual maps from three reference families through shared markers. Lastly, the markers falling into ‘zero recombination clusters’ that were excluded during map construction were anchored to the linkage map, based on the positions of their corresponding representative markers. All genetic linkage maps were drawn using MAPCHART 2.2.^37^

### 2.4. Differences in recombination rates between families and sexes

To assess differences in recombination rates among the three resource families, the sex-averaged map was used following the M-test according to Ott's method.^38^

$$X^{2}=2\times \text{ln(10)}\left[\sum Z_{i}({\hat{\theta }}_{i})-Z(\hat{\theta })\right]$$

where $Z_{i}({\hat{\theta }}_{i})$ represents the LOD scores of maximum-likelihood estimation (MLE) for the *i*th reference family for common marker pairs among the three families. The $Z(\hat{\theta })$ represents the total LOD scores of MLE for all three families. The recombination fractions for all mapped locus intervals from each family were obtained from JoinMap.

To investigate sex-specific heterogeneity throughout each linkage group, common marker pairs were used to compare the locus intervals across the male-specific map and female-specific map using contingency G-test.^39^

### 2.5. Integration of linkage map with physical map

To integrate previously developed physical map^40^ with the high-density linkage map constructed in the present work, all the mapped SNPs with 70-bp flanking sequences were aligned with BES^8,40^ and BAC-based physical map contig-specific sequences (PMCSS)^41^ using BLAST with the *E*-value cut-off 1E−10, minimal alignment length of 36, and minimal identity of 95%. The relationship of physical map and genetic map was built up based on the SNP-associated BES and PMCSS. The BAC physical map contigs that harboured BAC-derived BES and PMCSS were anchored onto the genetic map based on the SNP positions.

## 3. Results

### 3.1. Selection of SNP markers

As summarized in Table 1, SNP genotypes were obtained from 576 samples of three mapping families (192 samples per family). According to the assessment of genotyping quality and polymorphism in all samples from the three mapping families, genotypes of a total of 121,521 SNPs were used (Table 1). Owing to the low genotype calling rate (<95%), nine individuals were excluded for analysis, including one individual from family 1, three individuals from family 2, and five individuals from family 3. After further filtering to remove SNPs with low calling rate (<97%) and non-Mendelian inheritance (*P* < 0.001), a total of 18,866 SNPs from family 1, 33,224 SNPs from family 2, and 35,093 SNPs from family 3 were retained for linkage mapping. Taken together, a total of 64,889 SNPs that were informative in at least one of the three mapping families were used.

**Table 1.** SNPs selected for linkage mapping

Item	Number
SNPs on array	250,113
SNPs polymorphic and with high genotyping quality	121,521
SNPs used for linkage mapping after further filtering	64,889
SNPs from family 1 used for linkage mapping	18,866
SNPs from family 2 used for linkage mapping	33,224
SNPs from family 3 used for linkage mapping	35,093

### 3.2. Linkage mapping

Linkage maps were first constructed for each of the three families, separately. For all three mapping families, 29 linkage groups (LGs) were obtained, which was consistent with the number of chromosomes of the catfish haploid genome. The consensus genetic linkage maps were obtained by merging separate maps from three mapping families. A total of 31,387 markers with distinct genetic positions (hereafter referred to as unique markers) were placed on the consensus linkage maps, which included 8,644 SNPs from family 1, 13,477 SNPs from family 2, and 14,343 SNPs from family 3 (Table 2). After anchoring the previously excluded markers that fell into ‘zero recombination clusters’ based on the genetic positions of representative markers, a total of 54,342 SNP markers were placed onto the current linkage map (Table 2).

**Table 2.** SNPs placed on the linkage maps

Item	Number
Unique SNPs from family 1 mapped to linkage map	8,644
Unique SNPs from family 2 mapped to linkage map	13,477
Unique SNPs from family 3 mapped to linkage map	14,343
Total unique SNPs mapped to linkage map	31,387
Total SNPs mapped to linkage map	54,342

**Figure 1.**
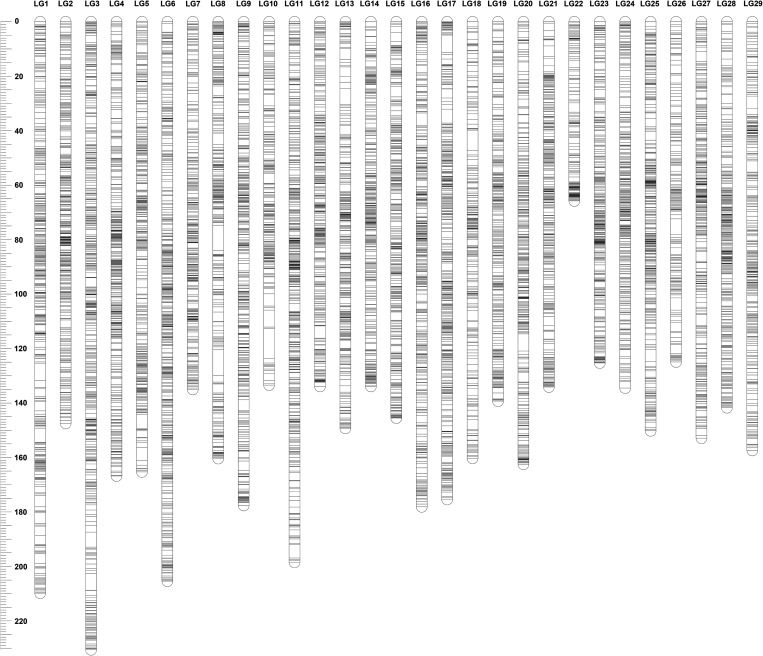
Illustration of female-specific linkage map.

**Figure 2.**
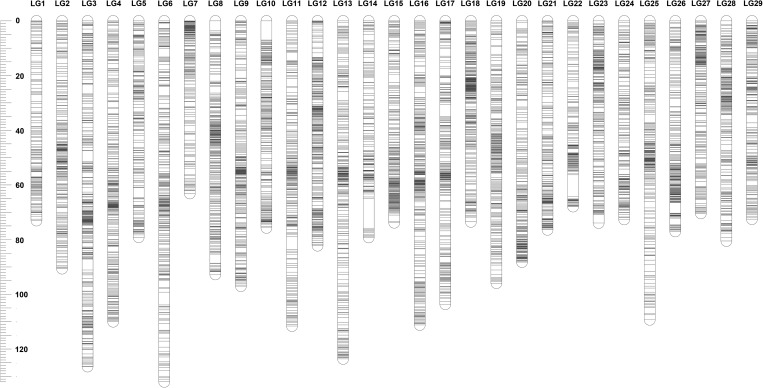
Illustration of male-specific linkage map.

The sex-specific maps were constructed using markers that were heterozygous in only female or male parent. The female genetic map consisted of 18,444 SNPs including 9,746 unique markers, with a total genetic length of 4,495.1 cM (Table 3; Fig. 1). The marker intervals estimated based on the unique marker positions ranged from 0.37 cM/marker pair in LG12 to 0.7 cM/marker pair in LG18 with the average marker interval of 0.46 cM/marker pair in female genetic map. The male genetic map comprised 15,148 SNPs with 7,250 unique markers and a total genetic length of 2,593.7 cM, which was much shorter than female genetic map. The marker intervals of male genetic map ranged from 0.25 cM/marker pair (LG18) to 0.55 cM/marker pair (LG1), with an average marker interval of 0.36 cM (Table 3, Fig. 2). It should be noted that these distances refer to intervals where recombination was detected and of course it should be recognized that in both sexes, the minimum distance observed was 0 cM for completely linked markers. The female- and male-specific maps are illustrated in Figs 1 and 2, respectively. The detailed map information was provided in Supplementary data S1.

**Table 3.** Summary of the sex-specific linkage maps of channel catfish

Linkage group	Female-specific map				Male-specific map				F:M ratio
	Mapped markers	Distinct positions	Genetic length (cM)	Marker interval (cM)	Mapped markers	Distinct positions	Genetic length (cM)	Marker interval (cM)	
1	810	397	209.8	0.53	374	153	83.9	0.55	2.50
2	616	370	147.1	0.40	517	251	90.6	0.36	1.62
3	934	481	230.6	0.48	665	342	126.4	0.37	1.82
4	774	380	166.8	0.44	699	313	110	0.35	1.52
5	643	353	165.3	0.47	441	199	79.1	0.40	2.09
6	821	470	205.3	0.44	606	300	132.1	0.44	1.55
7	583	313	134.9	0.43	370	196	63.1	0.32	2.14
8	550	326	160.4	0.49	615	297	92.7	0.31	1.73
9	751	399	177.6	0.45	546	291	97.1	0.33	1.83
10	488	238	133.4	0.56	516	228	75.7	0.33	1.76
11	815	434	198.5	0.46	616	273	111.6	0.41	1.78
12	695	361	133.9	0.37	727	311	82.2	0.26	1.63
13	604	311	149.3	0.48	557	285	123.6	0.43	1.21
14	598	340	133.9	0.39	351	150	79.2	0.53	1.69
15	651	332	145.5	0.44	524	248	73.8	0.30	1.97
16	799	385	178.1	0.46	635	337	111.2	0.33	1.60
17	792	420	175.4	0.42	506	264	103.6	0.39	1.69
18	473	230	160.3	0.70	640	290	73.5	0.25	2.18
19	542	302	139.3	0.46	495	256	95.9	0.37	1.45
20	657	344	162.5	0.47	557	244	88.2	0.36	1.84
21	576	318	134.1	0.42	437	217	76.4	0.35	1.76
22	308	153	65.8	0.43	380	169	67.9	0.40	0.97
23	668	319	125.3	0.39	478	245	73.9	0.30	1.70
24	518	296	134.5	0.45	408	195	72.6	0.37	1.85
25	665	348	150.2	0.43	535	269	109.3	0.41	1.37
26	389	181	124.9	0.69	512	242	76.9	0.32	1.62
27	595	327	152.9	0.47	522	233	70.4	0.30	2.17
28	663	334	141.7	0.42	484	237	80.5	0.34	1.76
29	466	284	157.4	0.55	435	215	72.5	0.34	2.17
Total	18,444	9,746	4,495.1	0.46	15,148	7,250	2,593.7	0.36	1.73

**Figure 3.**
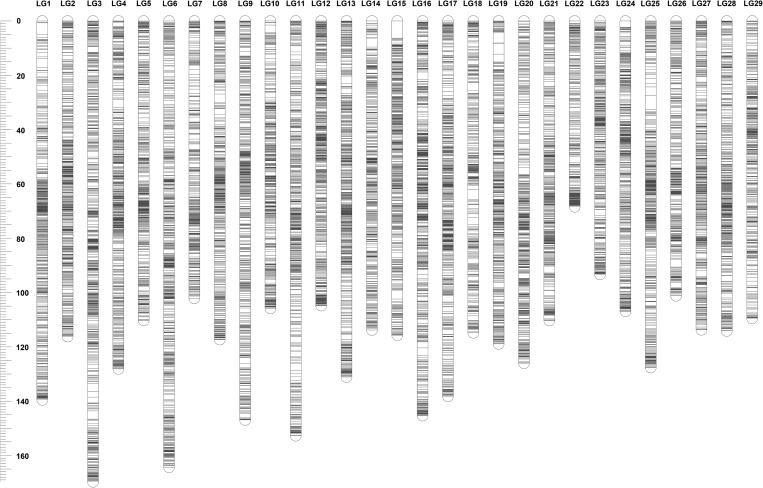
Illustration of sex-averaged linkage map.

The sex-averaged map was constructed using markers that were heterozygous in both parents. As summarized in Table 4, a total of 29,081 SNPs were mapped, which consisted of 15,598 unique markers. The sex-averaged map spanned 3,505.4 cM with an average marker interval of 0.22 cM, ranging from 0.17 cM/marker (LG12) to 0.30 cM/marker (LG18). The genetic map is illustrated in Fig. 3. The detailed map information was provided in Supplementary data S1.

**Table 4.** Summary of the sex-averaged linkage map of channel catfish

Linkage group	Sex-averaged map			
	Mapped markers	Distinct positions	Genetic length (cM)	Marker interval (cM)
1	1,039	556	139.5	0.25
2	1,014	557	116.2	0.21
3	1,212	673	169.7	0.25
4	1,220	653	128.1	0.20
5	1,002	491	110.2	0.22
6	1,183	680	163.3	0.24
7	861	422	102.1	0.24
8	1,073	557	117.3	0.21
9	1,100	604	146.9	0.24
10	913	475	105.8	0.22
11	999	572	152.7	0.27
12	1,216	625	104.8	0.17
13	1,176	630	131.1	0.21
14	868	472	113.8	0.24
15	1,047	562	115.7	0.21
16	1,219	695	145.3	0.21
17	1,178	623	138.2	0.22
18	782	388	114.8	0.30
19	914	523	118.9	0.23
20	1,029	550	126	0.23
21	968	484	110.3	0.23
22	779	364	68.4	0.19
23	929	486	93.3	0.19
24	854	492	106.9	0.22
25	1,097	584	127.6	0.22
26	811	427	101.2	0.24
27	925	516	113.7	0.22
28	969	516	114.1	0.22
29	704	421	109.5	0.26
Total	29,081	15,598	3,505.4	0.22

### 3.3. Analysis of recombination rates

**Figure 4.**
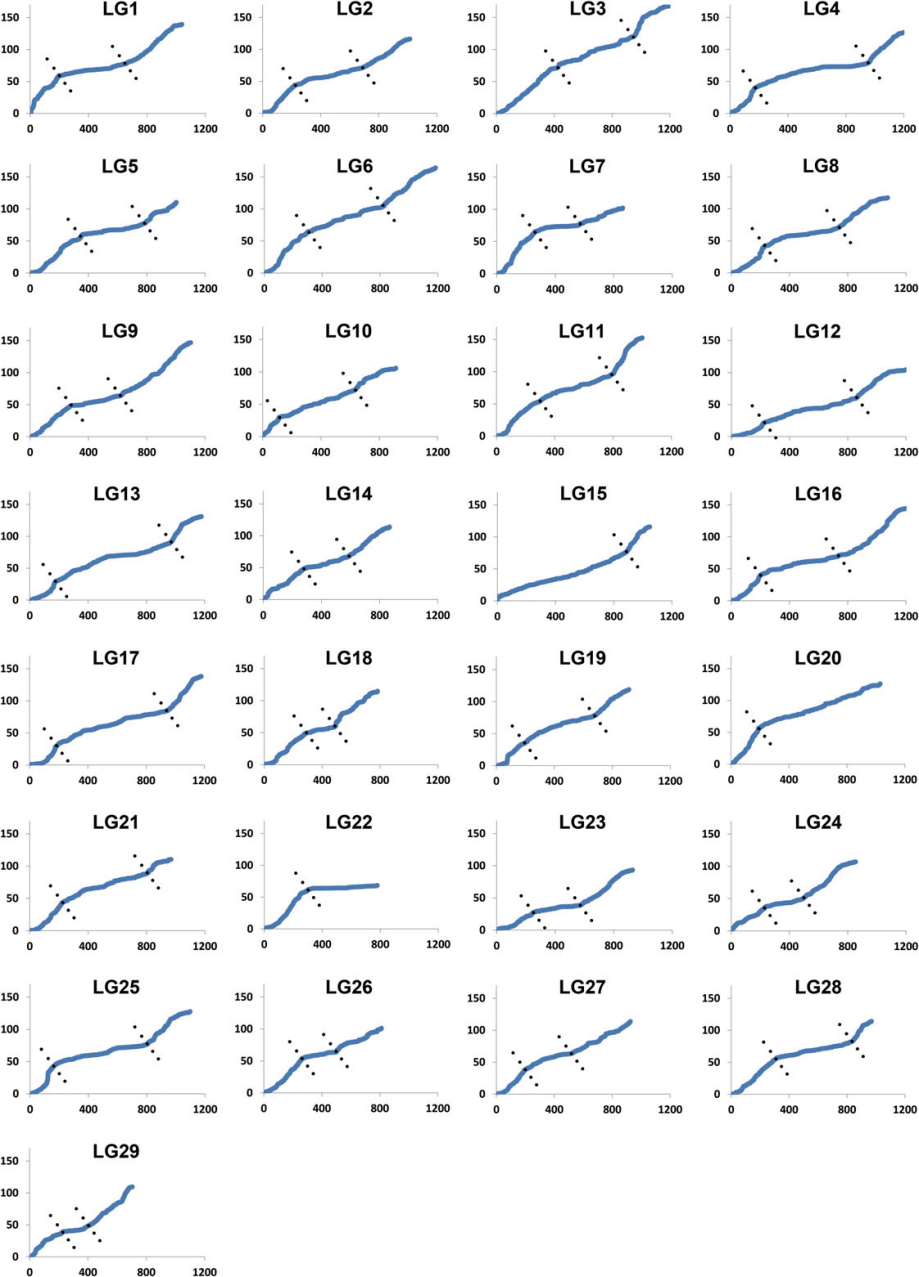
The patterns of localized regional recombination rates along each linkage group.

Within each linkage group, mild to strong localized specific recombination patterns were observed, whereby recombination rates were usually elevated towards the end and suppressed in the middle of the map (Fig. 4). Clustered markers that fell into ‘zero recombination rate’ were observed in every linkage group, especially in positions close to the centromeres in contrast to the telomeres (Supplementary data S1).

**Figure 5.**
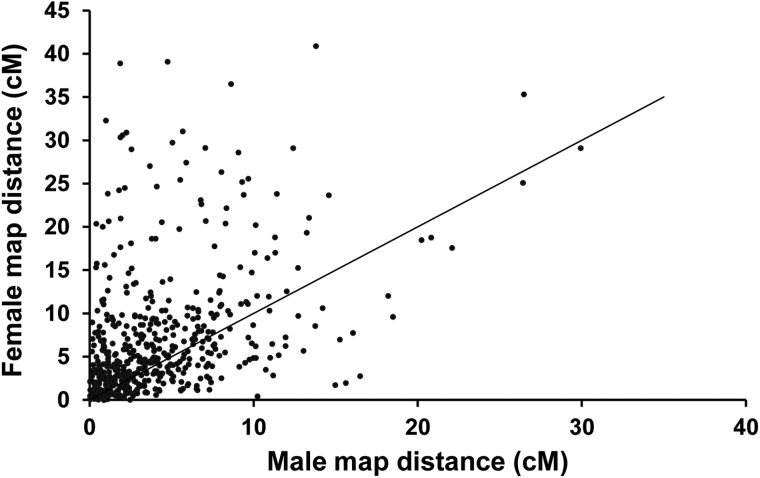
Comparison of the recombination rate between female and male. The inter-marker distances (cM) for all pairs of adjacent markers from both female- and male-specific maps were used. The diagonal line represents sex-equal recombination rates.

There is no significant difference in recombination rate among three mapping families based on the examination with common marker pairs among the three families (see Methods section). In contrast, significant higher recombination rates were observed in a majority of the linkage groups of the female genetic map than that of male genetic map (*P* < 0.01). Overall, the female genetic map was 1,901.4 cM longer than male genetic map, with an average female-to-male ratio of 1.7 : 1 (Table 3). The ratio varied by linkage groups, ranging from 0.97 : 1 in LG22 to 2.50 : 1 in LG1 (Table 3). The largest differences in recombination rate between the female and male maps were observed in LG1, LG5, LG7, LG18, LG27, and LG29. Across all these linkage groups, the female : male recombination ratios exceeded 2.0. LG22 was unusual in that recombination rates were very similar between the sexes (i.e. 0.97 : 1). Within unique map positions, 504 SNPs were commonly shared between the two sexes, while significantly higher recombination rates were observed in majority of the common locus intervals (*P* < 0.01) (Fig. 5).

### 3.4. Integration and validation with physical map

The integration of the genetic map with the BAC-based physical map anchored 2,728 (83%) of the 3,307 physical map contigs (Table 5). Together with the 1,481 (44.8%) physical map contigs that were anchored previously,^6^ we were able to anchor a total of 2,880 (87.1%) physical map contigs consisting of 28,416 BAC clones (92.9% of total BAC clones). The sizes of anchored physical map contigs varied from 66.0 kb to 2,005.8 kb (Supplementary data S2), with an average size of 301 kb. Together, a total of 867.4 Mb of the physical map was integrated by genetic linkage map that accounted for ∼90% of the channel catfish genome (Table 5). Detailed information regarding integration of linkage map and physical map is provided in Supplementary data S2.

**Table 5.** Integration of channel catfish linkage map with physical map

Item	Number	Percentage
Number of physical map contigs^a^	3,307	100
Number of physical map contigs with SNP markers	3,243	98.1
Number of physical map contigs anchored to linkage map in this work	2,728	82.5
Number of physical map contigs anchored in previous study^^b^^	1,481	44.8
Total physical map contigs mapped to linkage map	2,880	87.1
Total BAC clones in the catfish physical map^a^	30,582	100
Total BAC clones mapped on the linkage map	28,416	92.9
Total size of physical map contigs^a^	965,279	100
Total size of anchored physical map contigs	867,364	89.9

a Data obtained from Xu *et al.*^6^

b Data obtained from Ninwichian *et al.*^9^

**Figure 6.**
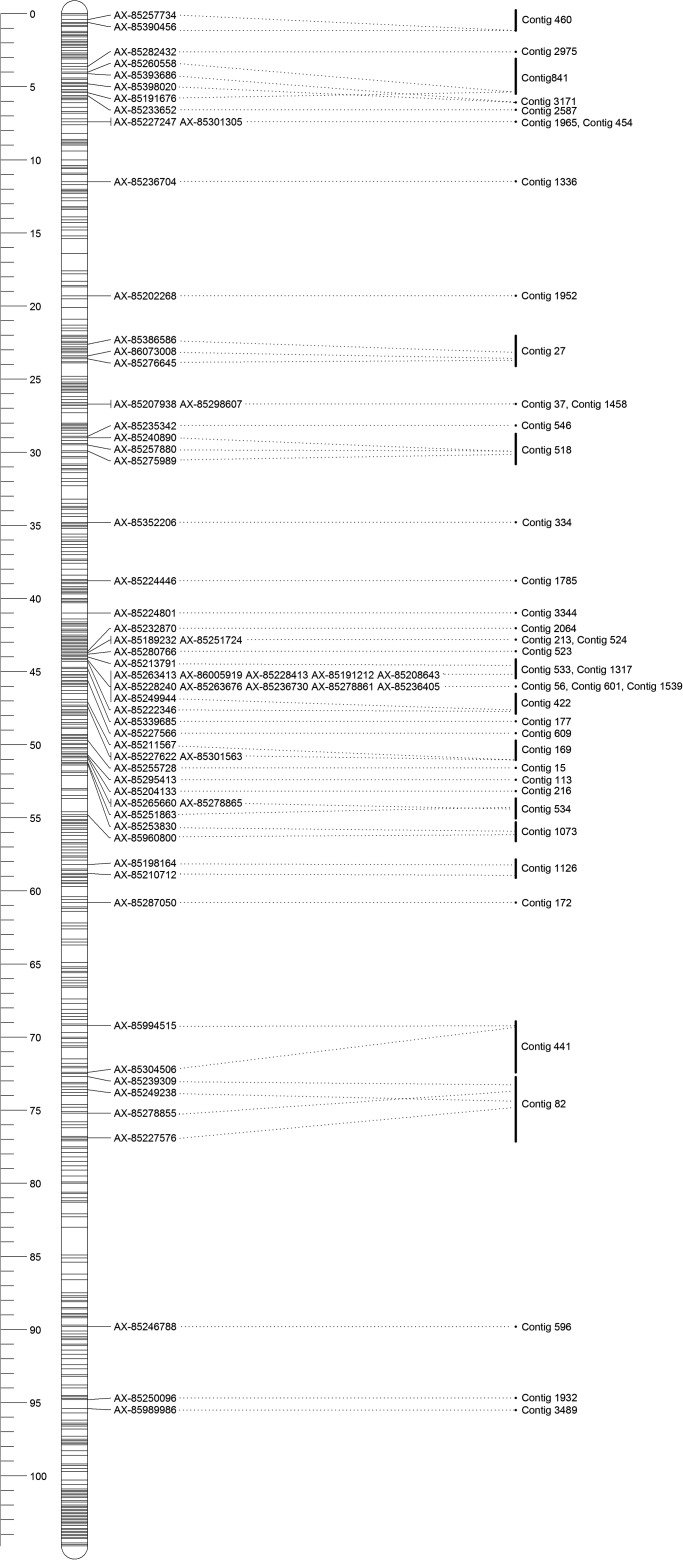
Illustration of integration of the linkage map (left) with the physical map (right). The linkage group 12 (LG12) was used for the illustration. Vertical bars represent physical map contigs containing at least two SNPs mapped onto the linkage map, and the dot spots indicate physical map contigs with only one SNP.

The integration of linkage map and physical map enabled the cross-validation of the quality of the physical map and the genetic map (Table 6). A total of 1,467 physical map contigs containing 2,961 BES-associated SNPs were placed onto linkage maps. With 615 physical map contigs, two or more SNP markers were mapped that allowed the determination of orientation of the physical map contigs along the chromosome. With such contigs, the quality of physical map contigs was assessed based on the mapped markers. An illustration with linkage group 12 is provided in Fig. 6. The vast majority of physical contigs were validated, but a small fraction of physical map contigs was found incorrectly assembled, or the ordering of the SNP markers onto the map was in error.

**Table 6.** Cross-validation between linkage map and physical map

Item	Number
Number of physical map contigs with BES-associated SNPs	2,790
Number of physical map contigs anchored through BES-associated SNPs	1,467
Number of physical map contigs with at least two BES-associated SNPs	615
Number of physical map contigs mapped onto different LGs	47
Number of BAC clones with BES-associated SNPs	2,707
Number of BAC clones with at least two BES-associated SNPs	238
Number of BAC clones mapped onto different LGs	7

**Figure 7.**
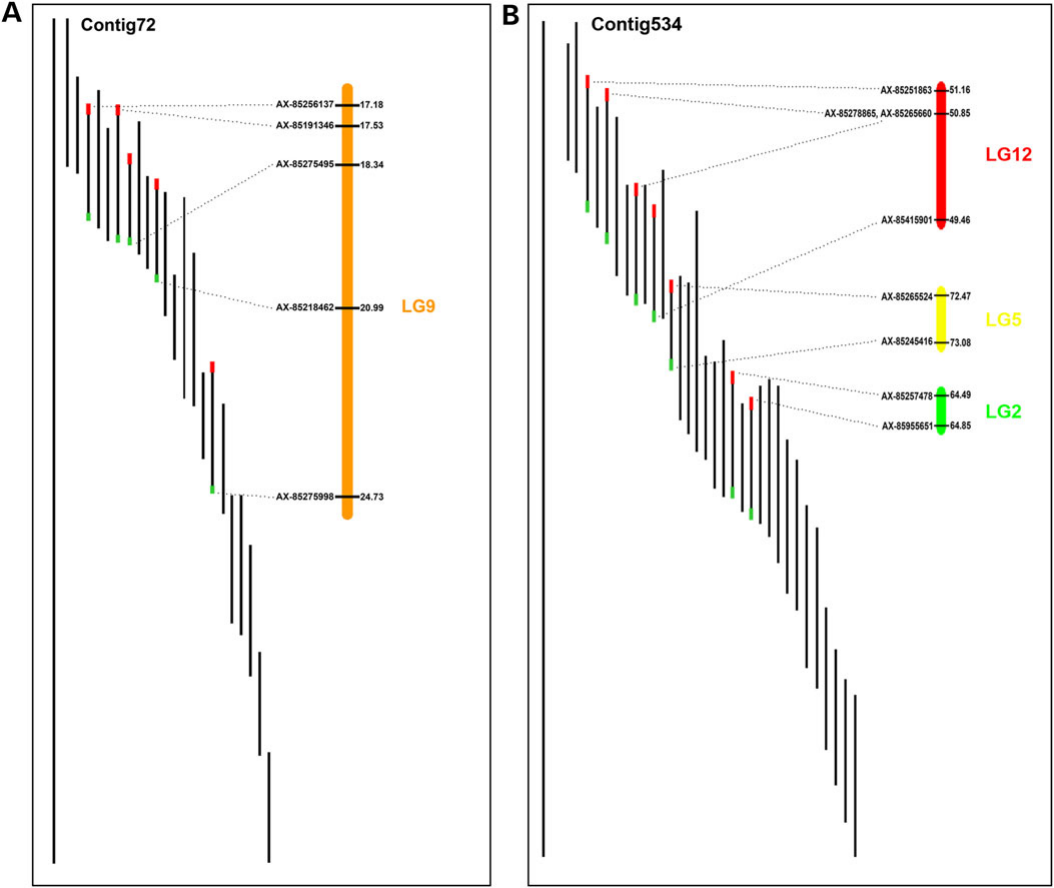
Example of validation of physical map using the linkage map. (A) shows the correct physical map contig using Contig72 as an example; (B) shows the incorrect physical map contig using Contig534 as an example.

A total of 47 physical map contigs had markers that were mapped in different linkage groups, indicating the potential errors of the physical map assembly or the mapping errors of incorrectly assigned SNPs. These errors could be caused by SNPs falling into duplicated regions of genome. Moreover, of the 2,707 BAC clones with BES-associated SNPs, 238 BAC clones contained at least two BES-associated SNPs. Within this group, 7 BAC clones were mapped onto different LGs, suggesting the misplacement of those SNP markers onto the linkage groups. As an illustration, the integration of sex-averaged linkage map with physical map Contig72 and Contig534 is shown in Fig. 7, presenting the correct and incorrect physical map assembly, respectively. The manual check of the potential assembly or mapping error warrants the correctness of both physical map and linkage map in the next step.

Physical distances were estimated based on the position of SNP markers within each physical map contig, which allowed for estimation of the ratios of physical distance to genetic distance (Supplementary data S3). On the other hand, recombination frequencies can be calculated for physical map contigs with multiple SNP markers mapped onto the linkage map. With the sex-averaged linkage map, the genetic size of 3,505.4 cM and the physical size of 965,279 kb based on the physical map, we estimated the average genetic size as 3.6 cM per Mb. The ratios observed from the 250 physical map contigs that contained multiple SNPs mapped onto the linkage map ranged from 0 to 2.26 cM per Mb. The markers with higher ratios might indicate the potential loci of recombination hot spots.

## 4. Discussion

In this work, we constructed a high-density genetic map for channel catfish. This map possesses the highest marker density among all the genetic linkage maps constructed for any aquaculture species. Taking advantage of the catfish 250K SNP array, we were able to efficiently and cost-effectively genotype 250,000 SNPs in the 576 fish from three mapping families (192 fish per family). Such a high-density linkage map should be a valuable resource for analysis and fine-scale QTL mapping in catfish.

Although the efficient SNP genotyping technology allowed the construction of high-density linkage map, the high-resolution was yet to be achieved. A large number of markers fell into ‘zero recombination clusters’ where no recombination events occur during meiosis between the markers among the fish used for mapping analysis. These represent closely arrayed adjacent SNP positions, and their ordering is likely best achieved by assessing their order within the aligned physical map contigs. Conversely, it was also observed that genetic recombination rates could be artificially inflated when genotyping errors occur or when markers with large amounts of missing data are included in the analysis.^42,43^ Similar situations were observed previously in zebrafish during the construction of high-density genetic map. In zebrafish, the genetic sizes of initial map were over 1,000 cM per chromosome. After removal of genotyping errors, the genetic sizes were reduced to around 100 cM.^44^ To reduce the effect of clustered markers on the map construction and reduce the computing load, we picked one representative anchor marker that had the most informative meiosis from each of these clusters in the linkage map construction. Afterwards, we rejoined the markers within the ‘zero recombination clusters’ that were excluded during the initial mapping steps. This procedure relocated the informative markers onto the linkage maps based on the positions of their representative anchor marker. Such clustered markers are still valuable for the chromosome-scale scaffolding.

With the availability of such high-density mapped SNPs, the patterns of marker distribution across chromosomes can be examined. It appears that clustered markers were commonly found in regions around centromeres while less frequently found around the telomeres. This was consistent with observations in various genetic maps previously developed in fish species, such as tilapia,^45^ medaka,^46^ rainbow trout,^47^ Atlantic salmon,^12^ and catfish.^9,16^ One potential explanation is that the centromeres contain abundant tandemly repeated, heterochromatic DNA sequences.^48^ As shown in Fig. 4, the distribution of mapped SNP markers across chromosomes in channel catfish is consistent with the observation of telomere and centromere effects,^49^ which would result in a higher recombination rate near the telomeres while a lower recombination rate near the centre of the chromosomes. The recombination rates might be positively correlated with GC content, as in human,^50^ pig,^51^ chicken,^52^ rodent,^53^ and yeast,^54^ or correlated with some other genomic features such as gene density and the presence of genes determining recombination hotspots. The PRDM9 gene was recently found to determine the recombination hotspots in the mammalian genomes.^55–57^ Future investigations on recombination landscape warrant the characterization of genomic features, affecting the meiotic recombination in catfish upon the availability of fully assembled genome sequences.

The suppression of recombination in males relative to the females observed in channel catfish (averaged female-to-male ratio is 1.7 : 1) was consistent with our previous observation^2^ as well as many other studies. Recombination rates differ between the two sexes in many organisms where recombination occurs more frequently in the homogametic sex than in the heterogametic sex.^50,58^ The similar phenomenon was reported in many other fish species such as Atlantic salmon (1.38 : 1), rainbow trout (1.68 : 1), European sea bass (1.60 : 1), Arctic char (1.69 : 1), and silver carp (1.52 : 1).^12,14,58–61^ Striking recombination differences between the sexes were observed in zebrafish (2.74 : 1)^58^ and grass carp (2.0 : 1).^62^ These observations indicated that even closely related fish species differ in genetic sizes and the extent of sexual dimorphism for recombination fraction,^50^ similarly as in mammals. Although the genetic basis for recombination bias between sexes remains unknown, several theories were proposed to explain this observation. One explanation from selection perspective suggests that selection pressure is stronger in male gametes than in female gametes during the haploid life stage. This difference could lead to male-specific selection to maintain beneficial haplotype to decrease the male recombination rate.^58,63^ An alternative explanation is that the female recombination rate is higher to compensate for the apparently less stringent checkpoint for achiasmatic chromosomes compared with males.^50^ In human, cytological studies suggested that sex-specific differences in recombination may derive from chromatin differences established prior to the onset of the recombination pathway.^64^

The high-density and the relatively high-resolution genetic map provides valuable resource for integrating the physical map and whole-genome assemblies. In this study, we anchored the catfish physical map to genetic linkage map through BES and PMCSS that contain SNP markers. With this linkage map of channel catfish, we were able to anchor 87% of the catfish BAC physical map contigs, covering ∼92% of the catfish genome. This is a great improvement on map integration compared with our previous efforts that only anchored 44.8% of the catfish BAC physical map contigs accounting for 52.8% of the catfish genome. To our knowledge, this is also the highest percentage of physical maps integration with genetic maps that were obtained in any aquaculture species. The well-integrated map is useful for comprehensive comparative genomic analyses, fine-scale mapping of QTL, and positional cloning for candidate genes.^10^ Moreover, the integrated map can be used as an important resource for the validation of the catfish whole-genome assembly.

## Supplementary data

Supplementary data are available at www.dnaresearch.oxfordjournals.org.

## Funding

This project was supported by Agriculture and Food Research Initiative Competitive Grant (No. 2012-67015-19410) from the US Department of Agriculture (USDA) National Institute of Food and Agriculture (NIFA) and partially supported by USDA Aquaculture Research Program Competitive Grant (No. 2014-70007-22395) from USDA NIFA. Funding to pay the Open Access publication charges for this article was provided by a grant from USDA Aquaculture Research Program Competitive Grant no. 2014-70007-22395.

## Supplementary Material

Supplementary Data
