# Supplementary material for: Construction of a high-density, high-resolution genetic map and its integration with BAC-based physical map in channel catfish
Source: DNA Res. 2014 Nov 26;22(1):39–52. doi: 10.1093/dnares/dsu038 (PMC4379976; doi:10.1093/dnares/dsu038)
Supplement: Supplementary Data [file supp_22_1_39__index.html]

Construction of a high-density, high-resolution genetic map and its integration with BAC-based physical map in channel catfish — Supplementary Data 

# Construction of a high-density, high-resolution genetic map and its integration with BAC-based physical map in channel catfish

## Supplementary Data

Supplementary Data

**Files in this Data Supplement:**

- Supplementary Data 1 - xlsx file
- Supplementary Data 2 - xlsx file
- Supplementary Data 3 - xlsx file
